# Chronic natriuretic peptide protein therapeutics

**DOI:** 10.1186/2050-6511-14-S1-O1

**Published:** 2013-08-29

**Authors:** Horng H Chen, Alessandro Cataliotti, John C Burnett

**Affiliations:** 1 Department of Medicine, Division of Cardiovascular Diseases, Mayo Clinic, Rochester, MN, USA

## 

The natriuretic peptides (NP) consist of a group of genetically distinct peptides with a 17 amino acid disulfide ring structure that exert important paracrine and endocrine actions in cardiorenal homeostasis. ANP and BNP bind to the natriuretic peptide-A receptor (NPR-A), which via 3',5'-cyclic guanosine monophosphate (cGMP) mediates natriuresis, vasodilatation, renin-aldosterone inhibition, anti-mitogenesis, and positive lusitropism. CNP lacks natriuretic actions, but possesses vasodilating and important growth- and fibrotic-inhibiting actions via the guanylyl cyclase-linked natriuretic peptide-B receptor (NPR-B). CD-NP now represents the first designer NP which, unlike ANP, BNP or CNP, co-activates both NPR-A and NPR-B.

In both experimental and human heart failure (HF), investigations have supported the hypothesis that the capacity of the cardiomyocytes to produce and/or release the NP may be overwhelmed in HF due to the increased demands of the system, thus leading to a state of relative deficiency of biologically active endogenous NP. Hence, giving the favorable cardiorenal and humoral proprieties of the NP, their chronic exogenous delivery is now viewed as favorable therapeutic opportunity for the treatment of cardiovascular diseases.

We have previously reported that subcutaneous (SQ) administration of BNP in experimental HF resulted in improved cardiac output with reduced systemic vascular resistance and cardiac filling pressures. Importantly, we translated these studies to humans and completed a pilot study to establish safety and efficacy of 8 weeks of chronic SQ BNP administration in human symptomatic systolic HF. More importantly, we have recently completed 2 proof of concept studies to assess the biological effects of 12 weeks of SQ BNP administration in patients with preclinical systolic dysfunction and patients with preclinical diastolic dysfunction (Stage B HF). These studies demonstrated that chronic SQ BNP induced a sustained generation of cGMP, which, importantly, was associated with persistent enhanced renal natriuretic response to volume overload.

Going beyond BNP, CD-NP (Cenderitide) is in clinical development as an outpatient therapy during the post-acute HF period, to be administered continuously for up to 90 days after hospital discharge via subcutaneous pump with the goals of reducing rehospitalization. Recently, a phase 1 study in HF patients was completed which demonstrated that SQ CD-NP infusion achieved desirable pharmacokinetic levels and was well tolerated. Pre-clinically, we have recently designed an in situ polymer precipitation delivery system suitable for the chronic and sustained release of CD-NP. With the appropriate polymer-gel formulation, CD-NP release could be sustained over 3 weeks, thus inducing prolonged cGMP activation and favourable biological actions.

Preclinical and early phase clinical studies have demonstrated the potential beneficial actions of chronic NP therapeutics. Further studies are warranted to determine if these functional responses can be translated into improved clinical outcomes and contribute to delaying the progression of HF.

